# Online Validation of a Battery of Questionnaires for the Assessment of Family Functioning and Related Factors

**DOI:** 10.3389/fpsyg.2020.00771

**Published:** 2020-04-28

**Authors:** Luigi Lavorgna, Marialaura Di Tella, Giuseppina Miele, Stefania Federica De Mercanti, Lidia Mislin Streito, Virginia Perutelli, Simona Bonavita, Lorys Castelli, Marinella Clerico

**Affiliations:** ^1^Department of Advanced Medical and Surgical Sciences, University of Campania Luigi Vanvitelli, Naples, Italy; ^2^Department of Psychology, University of Turin, Turin, Italy; ^3^II Clinic of Neurology, AOU University of Campania “Luigi Vanvitelli”, Naples, Italy; ^4^Department of Clinical and Biological Sciences, School of Medicine, University of Turin, AOU San Luigi Gonzaga, Turin, Italy

**Keywords:** online validation, family functioning, marital relationships, parental and peer attachment, alexithymia, psychological distress, social support

## Abstract

**Background:**

Family functioning relies on different factors that are related to the individual characteristics of each member, the social context in which the family nucleus is integrated, and the internal and interpersonal family factors. The Short Version of the Family Assessment Measure-III, Dyadic Adjustment Scale, Inventory of Parent and Peer Attachment, Twenty-item Toronto Alexithymia Scale, Hospital Anxiety and Depression Scale, and Multidimensional Scale of Perceived Social Support are among the most commonly employed self-report measures for the assessment of family functioning and related factors. Traditionally, these scales have been administered using paper-and-pencil versions. However, with increased access to the Internet, online administration of questionnaires has become more common. The present study aimed to validate an online version of each of the above-mentioned questionnaires in a heterogeneous sample of Italian healthy individuals.

**Methods:**

One-hundred participants were recruited for each questionnaire. A crossover design was used in each validation. The minimum important difference (MID) was applied to evaluate the differences in the variances of the paper-and-pencil and online format scores. A MID >0.5 is a reasonable first approximation of a threshold of important change. Taking into account the cross over design, mean difference between pencil-and-paper and online versions, and Intraclass Correlation Coefficient were also estimated by mixed models.

**Results:**

The MID was <0.5 for all the instruments used. Therefore, no significant difference was observed between the score variances of the paper-and-pencil and online formats of all the questionnaires. Moreover, for each questionnaire the difference between the means of online and paper-and-pencil administrations scores (mean O-P) was calculated. We reported 95% confidence intervals that did not include the 0; therefore, mean (O-P) was not statistically significant.

**Conclusions:**

The current findings indicate that the online versions of all the questionnaires we administered can be considered reliable tools for the assessment of family functioning and related factors.

## Introduction

Family is a complex, dynamic system that continuously evolves in search of a balance between the complementary tendencies of stability and transformation (Malagoli Togliatti and Catugno, 1996; Skinner et al., 2000; Pellerone et al., 2017). The functioning of this complex and delicate system relies on different factors, which are related with both the individual characteristics of each member and the social context in which the family nucleus is integrated, as well as with internal and interpersonal family factors, such as communication, cohesion, adaptability, quality of marital/parental–child relationships, and problem-solving abilities (Patterson and Garwick, 1994; McFarlane et al., 1995; Martinez and Forgatch, 2002; Walsh, 2003; Wood et al., 2017). Specifically concerning the internal factors, the Process Model of Family Functioning provides a conceptual framework to understand and assess these aspects within the family unit (Steinhauer et al., 1984). This model integrates several interrelated constructs (i.e., communication, affective expression, role performance, involvement, control, values, and norms) which facilitate the achievement of the main goal of the family, that is, the successful accomplishment of different developmental and crisis tasks (i.e., task accomplishment; Skinner et al., 2000). Each task requires the family to reorganize itself and to go through the following different phases to solve the problem: task identification, exploration of alternative solutions, implementation of selected approaches, and evaluation of effects. This model underlines that, through the process of task accomplishment, each family may meet the objectives central to its own life (Skinner et al., 2000). Skinner et al. (1983) tried to operationalize the seven constructs of the Process Model by developing the Family Assessment Measure (FAM). The FAM is an extensive tool designed for use in both clinical and research settings. At present, the third-edition of the FAM, conceived by Skinner et al. (2000) about 20 years later, is one of the most commonly employed self-report measures to evaluate family functioning.

Within the family system, interpersonal and relational factors, such as marital relationship and parental bonding, play a crucial role in determining adequate family functioning (Katz and Woodin, 2002; Parke, 2004). Family members influence each other both directly and indirectly (Minuchin, 2002). While on one hand, fathers and mothers can affect mother–child and father–child relationships, respectively, through their reciprocal interaction, on the other hand, children can indirectly influence the husband–wife relationship by modifying each parent’s behavior (Parke, 2004). Previous evidence highlighted that marital quality is linked with parent–child interactions and family well-being. For instance, happily married couples were found to show greater sensitivity, support, and warmth during family interactions as compared to unhappily married couples (Cowan et al., 1994; Minuchin, 2002).

Given the complexity of marital and parental relationships, different levels of analyses are necessary to understand the underlying family dynamics. Indeed, marital relationships and parental–child interactions require separate assessments to enable a holistic understanding (Parke and O’Neil, 1999). Among the different self-report instruments that have been developed to evaluate marital and parental–child relationships, the Dyadic Adjustment Scale (DAS; Spanier, 1976) and Inventory of Parent and Peer Attachment (IPPA; Greenberg et al., 1984; Armsden and Greenberg, 1987) are used most commonly. Particularly, the DAS has been designed to assess adjustment in all types of dyadic relationships, while the IPPA evaluates parental and peer attachment relationships in adolescents.

Regarding the individual characteristics of family members, personality traits are known to influence how each individual interacts with another (e.g., Asendorpf and Wilpers, 1998). Among these factors, growing evidence seems to show that difficulties in adequately recognizing one’s own emotions (i.e., alexithymia) are associated with a variety of interpersonal problems, including social isolation (e.g., Kokkonen et al., 2001), insecure attachment (Troisi et al., 2001), and maladaptive behaviors (Fonagy et al., 2002; Kooiman et al., 2004; Montebarocci et al., 2004; Besharat, 2010). Alexithymic individuals typically show limited capacity in processing emotional information, with resulting difficulties in identifying, understanding, and expressing their own feelings. These difficulties lead them to experience problems in dealing and communicating with other people, which in turn may cause increased levels of distress for the individuals themselves (Conrad et al., 2009; Besharat, 2010). This can affect family and social interactions negatively, with the possibility of destruction of marital and parental–child relationships. Assessing the presence of alexithymia and the level of psychological distress it may cause can thus help practitioners understand, more deeply, the individual characteristics that may interfere with adequate family functioning. The 20-item Toronto Alexithymia Scale (TAS-20; Taylor et al., 1985) is most commonly employed to assess alexithymia. The TAS-20 is a self-report questionnaire which examines the main features of alexithymia (Taylor et al., 2003). Concerning the assessment of psychological distress, different instruments have been developed to evaluate the levels of anxiety/depressive symptoms in both healthy and clinical populations (e.g., the Beck Depression Inventory, Beck et al., 1996; State-Trait Anxiety Inventory, Spielberger et al., 1983; Hospital Anxiety and Depression Scale – HADS, Zigmond and Snaith, 1983). Among these measures, the HADS is used most commonly, especially in clinical settings.

The last aspect to be considered in the assessment of family functioning is the social context in which the family unit is located and integrated. Within the social context, people are often connected with each other by different types of relations, to form a so-called social network (Wellman, 1981). The social network usually provides individuals with support and help, such as verbal and non-verbal advice, tangible aid, and emotional comfort, either in everyday situations or emergency circumstances (Stokes, 1983). The social support, provided by the social network through this emotional, informational, and instrumental assistance, usually has beneficial emotional or behavioral effects on recipients (Gottlieb, 1983; House and Kahn, 1985). Over the past decades, growing evidence has shown that social ties and social support are positively and causally related to individuals’ mental and physical health, and longevity (Seeman, 1996; Cohen and Janicki-Deverts, 2009; Ertel et al., 2009; Umberson and Montez, 2010; Thoits, 2011). Within the family nucleus, the presence of adequate levels of social support can represent a valuable resource the family members can count on, especially in the case of extremely stressful situations, such as a serious chronic medical condition or disability of one of the members (Kazak et al., 1997; Pakenham and Bursnall, 2006; Jiang et al., 2015; Kissel and Nelson, 2016).

Among the different instruments designed to assess social support, the Multidimensional Scale of Perceived Social Support (MSPSS; Zimet et al., 1988) is employed most commonly. The MSPSS is a self-report questionnaire which has been designed to evaluate the adequacy of an individual’s social support from three specific sources: the family, friends, and a significant other (Zimet et al., 1988).

Taken together, all these factors can help clinicians and researchers understand the dynamics and functioning of families better. The above-mentioned questionnaires are valid instruments that can be administered easily to patients and healthy individuals. Traditionally, these questionnaires and, in general, self-report instruments, have been administered using paper-and-pencil versions.

However, with the increased access to the Internet, which has grown dramatically over the past two decades (Gwaltney et al., 2008), the online administration of scales and questionnaires (Nguyen et al., 2017) can prove advantageous because it enables clinicians and researchers to reach a large number of participants in a short time. Online versions of scales and questionnaires have also been shown to have higher compliance rates than paper-based versions (U.S. Department of Health and Human Services FDA Center for Drug Evaluation, and Research, 2006; Lavorgna et al., 2018). Moreover, online versions have been found to be equivalent to paper-and-pencil ones, and these two versions can thus be used interchangeably (Vallejo et al., 2007; Coons et al., 2009; Bishop et al., 2010). However, the migration of questionnaires and scales from a paper-and-pencil to an online version is considered as a modification of the instrument that requires evidence to confirm if the two modes of administration perform equally well to guarantee the same validity, reliability, and quality of data (Lavorgna et al., 2018). Therefore, the main aim of the present study was to validate an online version of each of the above-mentioned questionnaires and scales (i.e., the Brief FAM-III, DAS, IPPA, TAS-20, HADS, and MSPSS), as an accurate, reliable online tool to assess family functioning and related factors.

## Materials and Methods

### Participants and Procedure

Considering the sample size used in previous studies on validation of web-based questionnaires and scales, 100 participants for each questionnaire and scale were recruited (Lavorgna et al., 2018). In particular, we enrolled 100 participants who completed exclusively the DAS, 100 adolescent participants who completed the IPPA questionnaire, and 110 participants in total who filled in the HADS, MSPSS, FAM-III, and TAS-20 (in the latter case, 92 participants initially answered to all four questionnaires, so 10 more participants were recruited to reach the total size of 100). Moreover, a sample size of 100 allowed us to identify an Intraclass Correlation Coefficient (ICC) of 0.9 with a 95% confidence interval and an error of 8%.

The data for the online versions of the questionnaires and scales were collected using the Google Form service. Inclusion criteria were the same as those indicated in articles reporting the Italian validation of measure assessed (Bressi et al., 1996; Gentili et al., 2002; Prezza and Principato, 2002; San Martini et al., 2009; Iani et al., 2014; Pellerone et al., 2017).

Participants were recruited to represent individuals with different social and cultural backgrounds. Particularly, individuals were enrolled from the following contexts:

1. the editorial board of a national Italian newspaper (*Corriere della Sera*), based in Campania Region;
2. the staff of the Marcianise City Hall, Caserta, Italy;
3. the staff of an Italian electronic company (Erregame);
4. the administration staff of an Italian telematic university (*Pegaso*);
5. an Italian scout group (A.G.E.S.C.I.);
6. a church community;
7. students of University of Naples Federico II, Naples, Italy.

We asked to all members of these different contexts their availability and willingness to take part in the study. The percentage of acceptance was about 50–60%. People who could not participate in the study, had prior work or family commitments or were not on their work shift during the recruitment process. Nobody refused to take part for other reasons or because not interested.

The study was approved by the AOU San Luigi Gonzaga Ethics Committee (CE 81/2019/U, 24 July, 2019; protocol number 10899, 2 August, 2019) and was conducted in accordance with the Declaration of Helsinki. All the participants provided written informed consent to participate in the study.

### Measures

#### Sociodemographic Information

Participants were asked to provide the following sociodemographic information: gender, age, educational level, and years of living together/marriage (for participants who responded to the DAS).

#### Psychological Questionnaires

##### Short version of the family assessment measure – third edition

The Brief FAM-III is one of the most frequently used self-report instruments to assess family functioning (Steinhauer et al., 1984; Skinner et al., 2000; Pellerone et al., 2017). It consists of three modules: the “General Scale,” which evaluates the family as a system; the “Dyadic Relationships Scale,” which examines how each family member perceives his/her relationship with another member; and the “Self-Rating Scale,” which allows each person to rate his or her own functioning within the family. Each scale consists of 14 items that are rated using a 4-point Likert scale.

In the present study, only the Self-Rating Scale was administered. This scale has shown good internal consistency, with Cronbach alpha values ranging from 0.80 to 0.88 (Skinner et al., 2000; Pellerone et al., 2017). In line with these results, in our sample the Cronbach’s alphas were good for the Self-Rating Scale of the Brief FAM-III (α paper-and-pencil = 0.86; α online = 0.86).

##### Dyadic adjustment scale

The DAS is a relationship adjustment self-reported measure (Spanier, 1976; Gentili et al., 2002) that is divided into four subscales: “Dyadic Consensus,” which evaluates the degree to which the respondent agrees with his/her partner; “Dyadic Satisfaction,” which assesses the degree to which the respondent feels satisfied with his/her partner; “Dyadic Cohesion,” which measures the degree to which the respondent and his/her partner participate in activities together; and “Affectional Expression,” which examines the degree to which the respondent agrees with his/her partner regarding emotional affection.

The DAS has shown good internal consistency (Cronbach alpha scores: 0.70–0.95) and test–retest reliability (Carey et al., 1993). In line with these results, in our sample the Cronbach’s alphas were excellent for the DAS (α paper-and-pencil = 0.92; α online = 0.92).

##### Inventory of parent and peer attachment

The IPPA is a self-report scale that measures adolescents’ perceptions of their attachment to their parents and peers. The first version of this instrument was developed by Greenberg et al. (1984) for adolescents aged 12–19 years. It comprises two subscales, one assessing attachment with parents and the other with peers. Subsequently, Armsden and Greenberg (1989) proposed a revised version in which the parental scale was split into two identical versions assessing attachment with mothers and fathers, respectively. This revised version consists of 75 items, equally divided between the three forms (i.e., maternal, paternal, and peer).

The items provide a global security attachment score and those on the following three dimensions of the attachment relationship: “Trust,” which refers to adolescents’ trust that parents and peers understand and respect their needs and desires; “Communication,” which refers to adolescents’ perception that parents and peers are sensitive and responsive to their emotional states; and “Alienation,” which refers to adolescents’ feelings of isolation, anger, and detachment experienced in attachment relationships with parents and peers.

The scale has shown good internal consistency (Cronbach alpha scores: 0.87 for the maternal form, 0.89 for the paternal form, and 0.92, for the peer form) and test–retest reliability (Armsden and Greenberg, 1987). In line with these results, in our sample the Cronbach’s alphas were excellent for the IPPA (α paper-and-pencil = 0.94; α online = 0.94).

##### Twenty-item toronto alexithymia scale

The TAS-20 is a self-report instrument designed to assess alexithymia (Taylor et al., 1985; Bressi et al., 1996). The results provide a TAS-20 total score and three subscale scores that assess the following aspects of alexithymia: “Difficulty identifying feelings,” which measures the inability to identify specific emotions or to distinguish between emotions and the bodily sensations of emotional arousal; “Difficulty describing feelings,” which assesses the inability to verbalize one’s emotions to other people; and “Externally-oriented thinking,” which evaluates the tendency of individuals to focus their attention externally and not on the inner emotional experience (Taylor et al., 2003). The TAS-20 cut-off scores are as follows: ≤51 no alexithymia, 52–60 borderline alexithymia, ≥61 alexithymia.

The scale has shown good internal consistency (Cronbach’s alpha coefficients: ≥0.70) and test–retest reliability (Taylor et al., 2003). In line with these results, in our sample the Cronbach’s alphas were good for the TAS-20 (α paper-and-pencil = 0.88; α online = 0.87).

##### Hospital anxiety and depression scale

The HADS is self-report measure used to assess psychological distress in clinical populations (Zigmond and Snaith, 1983; Costantini et al., 1999; Castelli et al., 2009). The HADS has also been used widely as an effective tool to assess psychological distress in non-clinical populations (Brennan et al., 2010). It includes 14 items representing two subscales, anxiety (HADS-A) and depression (HADS-D). Each subscale consists of seven items that are rated on a 0–3 scale, with the total score ranging from 0 to 21. A score of eight or more suggests a clinically significant level of depression/anxiety symptoms.

The HADS has shown good concurrent validity, test–retest reliability, and internal consistency (Cronbach’s alpha scores = 0.82–0.90) (Bjelland et al., 2002). In line with these results, in our sample the Cronbach’s alphas were good for the HADS (α paper-and-pencil = 0.80; α online = 0.79).

##### Multidimensional scale of perceived social support

The MSPSS is self-report measure of perceived social support (Zimet et al., 1988; Prezza and Principato, 2002). It consists of 12 items that are scored on a 7-point Likert-type scale. A total score and three subscale scores can be derived: “Significant Other,” “Family,” and “Friends.” Higher scores are associated with higher levels of perceived social support.

The MSPSS has shown good internal consistency (Cronbach alpha scores: 0.87–0.94) and test–retest reliability (Osman et al., 2014). In line with these results, in our sample the Cronbach’s alphas were excellent for the MSPSS (α paper-and-pencil = 0.93; α online = 0.93).

### Statistical Analyses

A crossover design was used in each validation, in which half of the participants (Group A1, A2, A3, A4, A5, and A6) were randomly selected to complete the paper-and-pencil format and the other half (Group B1, B2, B3, B4, B5, B6) completed the online format (Time 1). After a time-lapse of 3 weeks, to avoid learning effects, the groups were reversed, and the participants in Group A completed the scale in the online format and those in Group B completed the paper-and-pencil format (Time 2).

To evaluate the differences in score variances between the paper-and-pencil and online formats, we used the method proposed by Guyatt et al. (2002) for the instruments completed twice by the same participants. Specifically, we applied the minimum important difference (MID), defined as “the smallest difference in score in the absence of trouble and change in a patient’s management” (Jaeschke et al., 1989). For instance, if variances in pre and post test scores have a change score of under 0.5, there is no important alteration (Guyatt et al., 2002). Therefore, MID > 0.5 is a reasonable first approximation of a threshold of important change. Taking into account the cross over design of the present study, mean difference between pencil-and-paper and online versions, and ICC were also estimated by mixed models. Mean difference, ICC, and their 95% confidence intervals (95% CI) were reported. Effect size was also provided. Finally, a correlation matrix of the relationship between the questionnaires’ dimensions for the paper-and-pencil and online procedures, separately, was computed.

## Results

Sociodemographic characteristics for each group of participants have been presented in Table 1.

**TABLE 1:** Sociodemographic characteristics of the participants.

	Minimum	Maximum	Mean	SD
**Brief FAM-III (N = 100)**				
Age	21	75	40	12
Education	5	19	14.69	2.85
**DAS (N = 100)**				
Age	27	66	44.99	9.89
Education	5	18	15.10	3.51
Years of living together or Marriage	2	39	19.36	9.49
**IPPA (N = 100)**				
Age	13	19	18.1	1.51
Education	8	13	12.53	0.97
**TAS-20 (N = 100)**				
Age	21	75	40.5	12.1
Education	5	19	14.47	2.97
**HADS (N = 100)**				
Age	21	75	40.22	12.03
Education	5	19	14.73	2.88
**MSPSS (N = 100)**				
Age	21	75	40	12
Education	5	19	14.49	2.94

*SD, standard deviation; Brief FAM-III, Short version of the Family Assessment Measure – Third Edition; DAS, Dyadic Adjustment Scale; IPPA, Inventory of Parent and Peer Attachment; TAS-20, 20-item Toronto Alexithymia Scale; HADS, Hospital Anxiety and Depression Scale; MSPSS, Multidimensional Scale of Perceived Social Support.*

In our main analysis, the MID was <0.5 for all the instruments (Table 2). Therefore, no significant difference was observed between the score variances of the paper-and-pencil and online formats of the Brief FAM-III, DAS, IPPA, TAS-20, HADS, and MSPSS.

**TABLE 2:** Variance difference between the questionnaires scores for the paper-and-pencil and online formats.

	Group A_1_		Group B_1_
**Brief FAM-III (N = 100)**			
σ^2^ time 1 (paper-and-pencil format)	20.42	σ^2^ time 1 (on-line format)	11.63
σ^2^ time 2 (on-line format)	20.21	σ^2^ time 1 (paper-and-pencil format)	11.72
MID	0.21		0.09
**DAS (N = 100)**			
σ^2^ time 1 (paper-and-pencil format)	272.85	σ^2^ time 1 (on-line format)	287.81
σ^2^ time 2 (on-line format)	272.72	σ^2^ time 1 (paper-and-pencil format)	287.91
MID	0.13		0.10
**IPPA (N = 100)**			
σ^2^ time 1 (paper-and-pencil format)	271.71	σ^2^ time 1 (on-line format)	263.29
σ^2^ time 2 (on-line format)	272.19	σ^2^ time 1 (paper-and-pencil format)	262.89
MID	0.48		0.40
**TAS-20 (N = 100)**			
σ^2^ time 1 (paper-and-pencil format)	103.89	σ^2^ time 1 (on-line format)	173.50
σ^2^ time 2 (on-line format)	104.35	σ^2^ time 1 (paper-and-pencil format)	173.13
MID	0.46		0.37
**HADS (N = 100)**			
σ^2^ time 1 (paper-and-pencil format)	27.89	σ^2^ time 1 (on-line format)	28.29
σ^2^ time 2 (on-line format)	27.90	σ^2^ time 1 (paper-and-pencil format)	28.75
MID	0.01		0.46
**MSPSS (N = 100)**			
σ^2^ time 1 (paper-and-pencil format)	102.58	σ^2^ time 1 (on-line format)	195.46
σ^2^ time 2 (on-line format)	102.82	σ^2^ time 1 (paper-and-pencil format)	195.63
MID	0.24		0.17

*MID, minimum important difference; Brief FAM-III, Short version of the Family Assessment Measure – Third Edition; DAS, Dyadic Adjustment Scale; IPPA, Inventory of Parent and Peer Attachment; TAS-20, 20-item Toronto Alexithymia Scale; HADS, Hospital Anxiety and Depression Scale; MSPSS, Multidimensional Scale of Perceived Social Support.*

In addition, for each of these questionnaires we calculated the difference between the means of online and paper-and-pencil administrations scores (mean O-P). We reported 95% CI that did not include the 0; therefore, mean (O-P) was not statistically significant. No statistically significant difference was thus observed between the paper-and-pencil and online formats. Effect size values also confirmed that there was no administration effect (Table 3).

**TABLE 3:** Mean difference (mean O-P) between scores for the online and paper-and-pencil questionnaires, and Interclass Correlation Coefficient (ICC) are reported.

	Mean (O-P)	95% CI	ICC	95% CI	ES
HADS	0.03	–0.17; 0.23	0.98	0.97; 0.99	0.04
MSPSS	0.22	–0.11; 0.55	0.99	0.99; 0.99	0.19
Brief FAM-III	–0.06	–0.27; 0.15	0.96	0.95; 0.97	–0.08
DAS	1.11	0.46; 1.76	0.98	0.97; 0.99	0.47
IPPA	–0.19	–0.47; 0.09	1.00	0.99; 1.00	–0.19
TAS-20	–0.15	–0.47; 0.17	0.99	0.99; 0.99	–0.13

*O, online procedure; P, paper-and-pencil procedure; CI, Confidence Intervals; ICC, Interclass Correlation Coefficient; ES, Effect Size; HADS, Hospital Anxiety and Depression Scale; MSPSS, Multidimensional Scale of Perceived Social Support; Brief FAM-III, Short version of the Family Assessment Measure – Third Edition; DAS, Dyadic Adjustment Scale; IPPA, Inventory of Parent and Peer Attachment; TAS-20, 20-item Toronto Alexithymia Scale.*

Finally, the correlation matrix showed no statistically significant results for each dimension of the questionnaires we administered in both paper-and-pencil and online formats (Supplementary Appendix A).

## Discussion

The present study aimed to validate the online version of the Brief FAM-III, DAS, IPPA, TAS-20, HADS, and MSPSS, to be used for assessing family functioning. The migration of questionnaires and scales from a paper-and-pencil to an online version is considered as a modification of an instrument that requires evidence to determine if the two administration formats perform equally well to guarantee the same validity, reliability, and quality of data (Lavorgna et al., 2018).

The results of our study showed no significant difference in the score variances of the paper-and-pencil and online formats of the Brief FAM-III, DAS, IPPA, TAS-20, HADS, and MSPSS. These findings were further supported using other analytic approaches, which showed no statistically significant difference for each of those questionnaires performed in online and paper-and-pencil formats. The online versions of these instruments can thus be considered reliable tools for the assessment of family functioning and related factors.

The questionnaires we validated represent an extensive battery that can allow clinicians and researchers to understand the relational dynamics of each family nucleus better. Indeed, family is a complex, dynamic system, the functioning of which relies on different factors such as individual characteristics of each member, the social context in which the family nucleus is integrated, and interpersonal family relationships (Patterson and Garwick, 1994; McFarlane et al., 1995; Martinez and Forgatch, 2002; Walsh, 2003; Wood et al., 2017). The Brief FAM-III, DAS, IPPA, TAS-20, HADS, and MSPSS represent valid instruments that can be employed to evaluate each of these factors to conduct an in-depth analysis of the features of family functioning.

The present study has some limitations. First, our sample was not representative of the general Italian population. Though we tried to enroll participants from different backgrounds, we could not include individuals from all the different Italian cultural contexts. Moreover, although we adopted the same procedure as the original validation articles of the measures we administered, we did not verify the validity of our results also on a clinical population. Therefore, further research should be conducted to replicate our findings with other clinical and non-clinical samples. Finally, future studies should also attempt to control for other sociodemographic variables that we could not control for in our study, such as occupation, socioeconomic status, and marital status.

Despite these limitations, our study was the first to validate the online versions of an extensive set of questionnaires to assess family functioning. We found evidence that the online versions of the Brief FAM-III, DAS, IPPA, TAS-20, HADS, and MSPSS represent accurate and reliable tools that could be easily administered to a large number of participants. A holistic assessment of the major factors that are related to family functioning can help clinicians and researchers understand family dynamics in clinical and non-clinical populations better.

## Data Availability Statement

The datasets generated for this study are available on request to the corresponding author.

## Ethics Statement

The study involving human participants was reviewed and approved by the AOU San Luigi Gonzaga Ethics Committee (CE 81/2019/U, 24 July, 2019; protocol number 10899, 2 August, 2019) and was conducted in accordance with the Declaration of Helsinki. All the participants provided written informed consent to participate in the study. Written informed consent to participate in this study was provided by the participants’ legal guardian/next of kin.

## Author Contributions

LL, SB, MC, and LC conceived and designed the study. LL, GM, SD, LS, and VP collected the data. LL, GM, and SB analyzed the data. LL, MD, LC, and MC interpreted the data. LL, MD, LC, and MC wrote the manuscript. All authors concluded the results, discussed and approved the final version of the manuscript.

## Conflict of Interest

The authors declare that the research was conducted in the absence of any commercial or financial relationships that could be construed as a potential conflict of interest.
