# Supplementary material for: Online Validation of a Battery of Questionnaires for the Assessment of Family Functioning and Related Factors
Source: Front Psychol. 2020 Apr 28;11:771. doi: 10.3389/fpsyg.2020.00771 (PMC7199646; doi:10.3389/fpsyg.2020.00771)
Supplement: Supplementary file 1 [file Table_1.DOCX]

**Appendix A.** Correlation matrix of the relationship between the questionnaires’ dimensions for the paper-and-pencil and online procedures. Alpha values for the paper-and-pencil and online administration formats are also shown.

|  | **N item** | **Mean (O-P)** | **95% CI** | **ICC** | **95% CI** | **Alpha P** | **Alpha O** | ***p**** |
| --- | --- | --- | --- | --- | --- | --- | --- | --- |
| **DAS** | 32 |  |  |  |  | 0.92 | 0.92 | 0.999 |
| Dyadic consensus | 13 | 0.11 | -0.04; 0.26 | 1 | 0.99; 1.00 | 0.89 | 0.88 | 0.689 |
| Dyadic satisfaction | 10 | 0.59 | 0.25; 0.93 | 0.93 | 0.89; 0.95 | 0.81 | 0.82 | 0.808 |
| Dyadic cohesion | 5 | 0.43 | 0.07; 0.79 | 0.86 | 0.81; 0.91 | 0.74 | 0.77 | 0.619 |
| Affectional Expression | 4 | -0.03 | -0.09; 0.03 | 0.99 | 0.99; 1.00 | 0.57 | 0.58 | 0.928 |
| **IPPA** | 75 |  |  |  |  | 0.94 | 0.94 | 0.999 |
| Mother trust | 10 | -0.01 | -0.04; 0.03 | 1 | 0.99; 1.00 | 0.82 | 0.77 | 0.271 |
| Mother communication** | 9 | 0 | - | 1 | - | 0.86 | 0.86 | 0.999 |
| Mother alienation | 6 | 0.01 | -0.01; 0.03 | 1 | 0.99; 1.00 | 0.69 | 0.7 | 0.891 |
| Mother | 25 | 0 | 0-03; 0.04 | 1 | 0.99; 1.00 | 0.91 | 0.91 | 0.999 |
| Father trust | 10 | 0 | -0.03; 0.03 | 1 | 0.99; 1.00 | 0.87 | 0.87 | 0.999 |
| Father communication | 9 | -0.01 | -0.05; 0.03 | 1 | 1.00; 1.00 | 0.89 | 0.89 | 0.999 |
| Father alienation** | 6 | 0 | - | 1 | - | 0.76 | 0.76 | 0.999 |
| Father | 25 | -0.01 | -0.08; 0.06 | 0.99 | 0.99; 1.00 | 0.94 | 0.94 | 0.999 |
| Peer trust | 10 | -0.04 | -0.16; 0.08 | 0.99 | 0.98; 0.99 | 0.91 | 0.91 | 0.999 |
| Peer communication | 8 | 0.02 | -0.13; 0.09 | 0.99 | 0.99; 1.00 | 0.85 | 0.86 | 0.763 |
| Peer alienation | 7 | -0.13 | -0.24; -0.02 | 0.99 | 0.98; 0.99 | 0.69 | 0.69 | 0.999 |
| Peer | 25 | 0.2 | -0.41; 0.03 | 0.99 | 0.98; 0.99 | 0.93 | 0.93 | 0.999 |
| **HADS** | 14 |  |  |  |  | 0.8 | 0.79 | 0.821 |
| Anxiety | 7 | 0.09 | -0.08; 0.26 | 0.96 | 0.94; 0.97 | 0.74 | 0.74 | 0.999 |
| Depression | 7 | -0.07 | -0.22; 0.08 | 0.96 | 0.95; 0.98 | 0.7 | 0.67 | 0.682 |
| **Brief FAM-III** | 14 |  |  |  |  | 0.85 | 0.86 | 0.75 |
| *** No Subscales *** | | | | | | | | |
| **TAS-20** | 20 |  |  |  |  | 0.88 | 0.87 | 0.705 |
| Difficulty identifying feelings | 7 | -0.17 | -0.29; -0.05 | 0.99 | 0.99; 1.00 | 0.86 | 0.86 | 0.999 |
| Difficulty describing feelings | 5 | -0.09 | -0.23; 0.05 | 0.98 | 0.97; 0.99 | 0.76 | 0.75 | 0.869 |
| Externally-oriented thinking | 8 | 0.1 | -0.11; 0.32 | 0.96 | 0.95; 0.98 | 0.6 | 0.57 | 0.751 |
| **MSPSS** | 12 |  |  |  |  | 0.93 | 0.93 | 0.999 |
| Significant Other | 4 | 0.15 | -0.02; 0.32 | 0.98 | 0.98; 0.99 | 0.89 | 0.87 | 0.521 |
| Family | 4 | 0.2 | 0.07; 0.33 | 0.99 | 0.98; 0.99 | 0.88 | 0.87 | 0.759 |
| Friends | 4 | -0.11 | -0.21; -0.003 | 0.99 | 0.99; 1.00 | 0.9 | 0.9 | 0.999 |

**p* value comes comparing the alpha values of the two versions applying the R package Cocron. **The values are identical on paper-and-pencil and online versions.

O = online procedure; P = paper-and-pencil procedure; DAS = Dyadic Adjustment Scale; IPPA = Inventory of Parent and Peer Attachment; HADS = Hospital Anxiety and Depression Scale; Brief FAM-III = Short version of the Family Assessment Measure - Third Edition; TAS-20 = 20-item Toronto Alexithymia Scale; MSPSS = Multidimensional Scale of Perceived Social Support.
